# Molecular Characterization and Antimicrobial Susceptibility of Nasal *Staphylococcus aureus* Isolates from a Chinese Medical College Campus

**DOI:** 10.1371/journal.pone.0027328

**Published:** 2011-11-17

**Authors:** Jimei Du, Chun Chen, Baixing Ding, Jinjing Tu, Zhiqiang Qin, Chris Parsons, Cassandra Salgado, Qiangjun Cai, Yulong Song, Qiyu Bao, Liming Zhang, Jingye Pan, Liangxing Wang, Fangyou Yu

**Affiliations:** 1 School of Laboratory Medicine, Wenzhou Medical College, Wenzhou, China; 2 Department of Respiratory Medicine, The First Affiliated Hospital of Wenzhou Medical College, Wenzhou, China; 3 Department of Intensive Care Unit, The First Affiliated Hospital of Wenzhou Medical College, Wenzhou, China; 4 Division of Infectious Diseases, Department of Medicine, Hollings Cancer Center, Medical University of South Carolina, Charleston, South Carolina, United States of America; 5 Department of Laboratory Medicine, The First Affiliated Hospital of Wenzhou Medical College, Wenzhou, China; 6 Department of Clinical Laboratory, Taizhou Center Hospital, Taizhou, China; University of Birmingham, United Kingdom

## Abstract

*Staphylococcus aureus* colonization and infection occur more commonly among persons living or working in crowded conditions, but characterization of *S. aureus* colonization within medical communities in China is lacking. A total of 144 (15.4%, 144/935) *S. aureus* isolates, including 28 (3.0%, 28/935) MRSA isolates, were recovered from the nares of 935 healthy human volunteers residing on a Chinese medical college campus. All *S. aureus* isolates were susceptible to vancomycin, quinupristin/dalfopristin and linezolid but the majority were resistant to penicillin (96.5%), ampicillin/sulbactam (83.3%) and trimethoprim/sulfamethoxazole (93.1%). 82%, (23/28) of the MRSA isolates and 66% (77/116) of the MSSA isolates were resistant to multiple antibiotics, and 3 MRSA isolates were resistant to mupirocin—an agent commonly used for nasal decolonization. 16 different sequence types (STs), as well as SCC*mec* genes II, III, IVd, and V, were represented among MRSA isolates. We also identified, for the first time, two novel STs (ST1778 and ST1779) and 5 novel *spa* types for MRSA. MRSA isolates were distributed in different sporadic clones, and ST59-MRSA-VId- t437 was found within 3 MRSA isolates. Moreover, one isolate with multidrug resistance belonging to ST398-MRSA-V- t571 associated with animal infections was identified, and 3 isolates distributed in three different clones harbored PVL genes. Collectively, these data indicate a high prevalence of nasal MRSA carriage and molecular heterogeneity of *S. aureus* isolates among persons residing on a Chinese medical college campus. Identification of epidemic MRSA clones associated with community infection supports the need for more effective infection control measures to reduce nasal carriage and prevent dissemination of MRSA to hospitalized patients and health care workers in this community.

## Introduction

*Staphylococcus aureus* is one of the most prevalent and clinically significant pathogens worldwide, causing a variety of illnesses ranging from benign, superficial skin eruptions to life-threatening infections with bacteraemia, endocarditis, pneumonia and toxic shock syndrome [1]. Since methicillin-resistant *S. aureus* (MRSA) was first identified in 1961, it has become the most common cause of nosocomial and community infections worldwide [2]. The recent emergence of highly virulent community-associated MRSA (CA-MRSA) isolates which cause disease in individuals with no apparent risk factors for hospital acquisition of MRSA has raised widespread concern over how CA-MRSA is transmitted in the community setting [3], [4].

*S. aureus* is a member of commensal microflora and readily colonizes the anterior nares in humans. Many infections caused by *S. aureus* occur in persons with prior nasal carriage [5], and this carriage is an important risk factor for nosocomial *S. aureus* infection in patients undergoing surgery, hemodialysis, implantation of intravascular devices, and among HIV-infected patients [6]. One study revealed that nasal carriage of *S. aureus* is an important source of *S. aureus* bacteremia [7], and eliminating nasal carriage of *S. aureus* may prevent systemic *S. aureus* infection [7]. Determination of the prevalence of *S. aureus* nasal carriage, as well as antimicrobial resistance profiles and molecular typing for nasal *S. aureus* isolates, in healthy populations is beneficial for identifying risk factors associated with *S. aureus* infection [8], [9], [10]. Molecular typing of *S. aureus* is also helpful for supporting infection control measures, investigating suspected outbreaks and preventing nosocomial transmission [11]. Identification of (Panton-Valentine Leukocidin) PVL genes, pulsed-field gel electrophoresis (PFGE), staphylococcal cassette chromosome *mec* (SCC*mec*) typing, spa typing, and multilocus sequence typing (MLST) have been used to monitor the evolutionary process of pandemic clones [12]. However, studies characterizing *S. aureus* isolates in China have largely focused on isolates recovered from clinical specimens in the context of clinical disease. One surveillance study performed in China showed that 63% of *S. aureus* isolates were MRSA, including 77% of nosocomial isolates and 43% of community isolates [13]. In addition, two major epidemic MRSA clones, ST239-MRSA-SCCmec type III and ST5-MRSA-SCCmec type II, are distributed across China in unique geographic patterns [14]. These data suggest that dissemination of virulent MRSA clones among healthy persons in China may contribute to the presence of clinically significant MRSA infections in some locations. To our knowledge, there are limited published studies characterizing MRSA isolates recovered from nares of healthy individuals in China who work and reside in the health care setting. Therefore, we sought to determine the prevalence, antimicrobial resistance profiles, and molecular characteristics of nasal *S. aureus* isolates from students on a large medical college campus in China.

## Materials and Methods

### Isolation and identification of nasal *S. aureus* isolates

935 volunteers from the campus of Wenzhou Medical College in Wenzhou, Southeast China without symptoms or signs of clinical illness were enrolled in the study over a one-month time period. The healthy volunteers included had not used antibiotics in past two months. Samples were collected from both anterior nares of volunteers by rotating a sterile polyester fiber-tipped swab moistened with sterile saline. Swabs were placed in 3 mL of Luria-Bertani broth and transported to the Department of Clinical Microbiology of the first Affiliated Hospital of Wenzhou Medical College. Gram-positive, catalase-positive, coagulase-positive isolates were confirmed as *S. aureus* using a Vitek-60 microbiology analyzer (bioMe'rieu, Marcy l'Etoile, France). The Ethics Committee of the first Affiliated Hospital of Wenzhou Medical College exempted this study from review because the present study focused on bacteria.

### Susceptibility testing

*S. aureus* susceptibility to penicillin (10 µg), ampicillin/sulbactam (20/10 µg), cefazolin (30 µg), vancomycin (30 µg), erythromycin (15 µg), clindamycin (2 µg), rifampicin (5 µg), linezolid (30 µg), mupirocin (5 µg), quinupristin/dalfopristin (15 µg), tetracycline (30 µg), trimethoprim/sulfamethoxazole (1.25/23.75 µg), gentamicin (10 µg), ciprofloxacin (5 µg), and levofloxacin (5 µg) were determined using the disk diffusion method in accordance with standards recommended by the Clinical and Laboratory Standards Institute (CLSI) [15]. All disks were obtained from Oxoid Ltd., and *S. aureus* ATCC 25923 was used as a quality control strain. Susceptibility of *S. aureus* to mupirocin was also determined using disk diffusion, with a zone diameter ≥14 mm using a 5-µg disc indicating susceptibility as described previously [16]. MICs of mupirocin for mupirocin-resistant isolates were further determined by the agar dilution method in accordance with the CLSI guidelines [15]. *mupA* was detected among mupirocin-resistant isolates as described previously[17].

### DNA Extraction

*S. aureus* isolates were cultured on blood agar overnight. Three to four bacterial colonies were suspended in 150 µL sterile distilled water with lysostaphin (1 mg/mL) (Sangon, China) and incubated at 37°C for 30 min. DNA was extracted using the Genomic DNA Extraction kit according to the manufacturer's instructions (Sangon, China). DNA was stored at −20°C and prepared for PCR amplification.

### Detection of MRSA and PVL genes

A multiplex PCR protocol described previously was used for simultaneous amplification of *mecA*, 16S rRNA, and PVL genes [18]. Isolates harboring *mecA* were confirmed as MRSA using MRSA N315 as a positive control strain.

### SCC*mec* typing

SCC*mec* typing of MRSA isolates was performed using eight unique and specific pairs of primers for SCC*mec* types and subtypes I, II, III, IVa, IVb, IVc, IVd, and V as described previously [19]. The MRSA isolates with unexpected fragments or lacking fragments by multiplex PCR were defined as non-typeable (NT). MRSA NCTC 10442 (SCC*mec*I), MRSA N315 (SCC*mec* II), MRSA 85/2082 (SCC*mec* III), MRSA JCSC 4744 (SCC*mec* IV) and MRSA WZ153 (SCC*mec* V) were used for positive controls.

### *spa* typing

The *spa* variable repeat region from each MRSA isolate was amplified by simplex PCR oligonucleotide primers as previously described [20], [21]. Purified *spa* PCR products were sequenced, and *spa* types were assigned by using the *spa* database website (http://www.ridom.de/spaserver).

### Multilocus sequence typing (MLST)

MLST of MRSA isolates was conducted through amplification of internal fragments of the seven housekeeping genes of *S. aureus* as described previously [22]. Following purification and sequencing of these genes, allele quantification and sequence typing were assigned using a well-characterized online database (http://saureus.mlst.net/). The DNA sequences of novel STs were verified by Pro. Enright and deposited in MLST database.

## Results

A total of 144 *S. aureus* isolates were isolated from the nares of the 935 volunteers in the study. The nasal carriage rate of *S. aureus* was 15.4% (144/935). 19.4% (28/144) of *S. aureus* isolates were identified as MRSA. The prevalence of nasal MRSA carriage was 3.0% (28/935). The resistance profiles of MRSA, MSSA and *S. aureus* isolates to antimicrobials tested were listed in Table 1. All *S. aureus* isolates tested were susceptible to vancomycin, quinupristin/dalfopristin and linezolid. The majority of *S. aureus* isolates were resistant to penicillin (96.5%), ampicillin/sulbactam (83.3%) and trimethoprim/sulfamethoxazole (93.1%). Isolates displayed complete or intermediate levels of resistance to gentamicin (16.0% and 18.1%), tetracycline (28.5% and 16.7%), ciprofloxacin (23.6% and 22.2%), clindamycin (31.2% and 23.6%) and erythromycin (52.1% and 30.6%). The resistance rates of MRSA isolates to gentamicin, ciprofloxacin, levofloxacin, erythromycin and clindamycin were significantly higher than those among MSSA isolates (Table 1). However, the intermediate resistance rates of MSSA isolates to gentamicin, tetracycline, erythromycin, clindamycin and rifampicin were higher than for MRSA isolates (Table 1). 82.1% (23/28) of MRSA isolates and 66.4% (77/116) of MSSA isolates were resistant to at least 3 different antimicrobials tested. Three MRSA isolates with no zone of inhibition to mupirocin (2.1%, 3/144) were resistant to mupirocin. The mupirocin MICs for the 3 MRSA isolates were >512 µg/mL determined by agar dilution method. The 3 MRSA isolates were positive for *mup*A detected by PCR.

**Table 1:** Antimicrobial susceptibility profiles of MRSA, MSSA and *S. aureus* nasal isolates.

	MRSA (n = 28)^a^		MSSA(n = 116)		*S. aureus*(n = 144)	
	R(%)	I(%)	R(%)	I(%)	R(%)	I(%)
penicillin	100		95.7		96.5	0
Ampicillin/Sulbactam	92.9		82.8		83.3	1.4
cefazolin	7.1	7.1	0.9	0	2.1	1.4
trimethoprim/sulfametho-xazole	85.7	0	94.8	0	93.1	0
gentamycin	28.6	7.1	12.9	20.7	16.0	18.1
tetracycline	28.6	0	28.4	20.7	28.5	16.7
vacomycin	0	0	0	0	0	0
ciprofloxacin	53.6	17.9	16.4	23.3	23.6	22.2
levofloxacin	39.3	17.9	6.9	6.0	13.2	8.3
erythromycin	75	14.3	46.6	34.5	52.1	30.6
clindamycin	53.6	10.7	25.9	27.6	31.2	23.6
rifampicin	3.6	0	6.9	8.6	6.3	6.9
Linezolid	0	0	0	0	0	0
dalfopristin/quinupristin	0	0	0	0	0	0
mupirocin	10.7	0	0	0	2.1	0

a : R, resistance; I, intermediate susceptibility.

MRSA isolates expressing SCC*mec* V (15 isolates), SCC*mec* II (5 isolates), SCC*mec* III (4 isolates) and SCC*mec* IVd (3 isolates) were identified (Table 2). The remaining isolate was non-typeable by multiplex PCR. MLST revealed 16 different sequence types (STs) for 22 of the MRSA isolates, including ST6, ST15, ST25, ST59, ST88, ST188, ST338, ST438,ST398, ST943, ST946, ST1295, ST1556, ST1623, ST1778 and ST1779 were identified (Table 1). Since at least one of the 7 housekeeping genes for MLST typing could not be amplified among the remaining 6 MRSA isolates, STs for these isolates were not available. The STs most commonly identified were ST59 (4 isolates), ST25 (3 isolates), ST188 (2 isolates) and ST438 (2 isolates). Two novel STs recently deposited in the MLST database (http://saureus.mlst.net/), ST1778 and ST1779, were also identified.

**Table 2:** Molecular characterization of MRSA nasal isolates and their antibiotic resistance profiles.

	SCC*mec*	spa type	STs	Resistance profile^a^	*pvl* b
N2	V	t437	1556	P,AML,CIP	+
N18	V	t163	338	P,AML,SXT,E,DA,TE,RD	+
N30	VId	t437	59	P,AML,STX,E,KE	−
N39	V	NT	438	P,AML,SXT,E,DA,CIP,LEV	−
N44	V	t289	943	P,AML,SXT,E	−
N54	V	new	88	P,AML,E,DA,CIP,LEV,CN,TE	−
N55	VId	t437	59	P,AML,E,DA	−
N57	V	t177	438	P,AML,SXT,E,DA,CIP,LEV,CN,TE	−
N58	II	new	NT^c^	P,AML,SXT,E, DA,CIP,CN,TE	−
N77	II	new	NT	P,AML,SXT,E,DA,CIP,LEV,CN,TE	−
N86	V	t571	398	P,AML,SXT,E,DA,CIP,CN	−
N91	III	t304	59	P,AML,SXT	−
N92	III	t304	NT	P,AML,SXT,E,DA,CIP,LEV, MUP	−
N97	VId	t437	59	P,AML,SXT,E,DE,TE	−
N98	V	new	946	P,AML,SXT,E,DA	−
N99	III	NT	6	P,SXT,DA	−
N108	V	t189	188	P,AML,SXT	−
N112	II	t081	NT	P,AML,SXT,E, DA, CIP,LEV	−
N114	V	t081	25	P,AML, SXT,E,CN,TE	−
N121	V	NT	1778	P,AML,SXT,E,DA,CIP,LEV,CN,TE	−
N123	V	NT	1623	P,AML,SXT,CIP,CN,KZ	−
N128	NT	t078	25	P,AML,SXT,E	+
N135	III	t062	1779	P,AML,E,CIP,LEV	−
N145	V	t1751	188	P,AML,SXT,E,CIP,LEV	−
N154	V	t078	25	P,AML	−
N156	V	new	1285	P,SXT	−
N167	II	t5196	15	P,AML, SXT,E,CIP,LEV	−
N168	II	t1751	NT	P,AML,SXT,E,DA,CIP,LEV,MUP	−

a P, penicillin; AML, Ampicillin/Sulbactam; CIP, ciprofloxacin; LEV, levofloxacin; SXT, trimethoprim/sulfamethoxazole; E, erythromycin; DA, clindamycin; RD, rifampicin; CN, gentamycin; KZ, cefazolin; TE, tetracycline; MUP, mupirocin.

b −, *pvl* negative; +, *pvl* positive.

c NT, not typeable.

19 MRSA isolates were assigned 12 previously characterized *spa* types, and 5 other isolates were assigned 5 novel *spa* types (Table 2). *spa* typing could not be assigned for the remaining 5 MRSA isolates. Furthermore, MRSA isolates were distributed among different sporadic clones. ST59-MRSA-VId-*spa* t437 was identified for 3 MRSA isolates. Three isolates, representing ST1556-MRSA-V- t437, ST25-MRSA-NT- t078 and ST338-MRSA-V- t163 clones, harbored PVL genes. One isolate exhibiting multidrug resistance was characterized as an ST398-MRSA-V-*spa* t571 clone. Three MRSA isolates typed as ST25 exhibited different spa types and SCC*mec* types and belonged to different clones. Although 2 ST188 MRSA isolates harbored SCC*mec* (V), these isolates exhibited two different *spa* types, t189 and t1751.

## Discussion

The prevalence of *S. aureus* nasal carriage among healthy adults ranges from approximately 20% to 30%, with higher prevalences in overcrowded populations [5], [6], [8], [23]. In the United States, a national population-based survey conducted over a 3-year period revealed nasal *S. aureus* colonization for 32% of study participants [24]. In contrast, a relatively low rate of nasal *S. aureus* carriage (6.3%) was found for children under the age of 5 in India [25]. The prevalence of nasal colonization with *S. aureus* in our study was 15.4%, a prevalence similar to that observed (16%) for a suburban military camp cohort in Beijing, China [26]. However, the prevalence of nasal MRSA carriage in our study (3.0%) was higher than prevalences previously reported for cohorts from developed countries, including the United States [24], [27], [28]. Furthermore, no nasal MRSA colonization was found among Chinese healthy military volunteers in another study [26]. In contrast, a relatively high prevalence of MRSA colonization was found (11.6%) for a cohort of healthy children aged ≤14 years in community settings in Taiwan over a 5-year period [29]. These data indicate that MRSA colonization varies significantly, even within similar populations in developed countries. The isolation of nasal *S. aureus* with a single sample may result in underestimating the number of intermittent carriers. In order to improving the isolation of nasal *S. aureus*, multiple nasal swabs should be taken from both anterior nares of volunteers. Additional studies are needed to clarify relationships between MRSA colonization, age, general antibiotic use, and other variables within overcrowded populations at higher risk for MRSA infections.

Our study revealed that 93% of nasal *S. aureus* isolates were resistant to trimethoprim-sulfamethoxazole, with MSSA isolates exhibiting the highest rates of resistance. These data contradict several studies reporting low rates of resistance to trimethoprim-sulfamethoxazole for *S. aureus* isolates recovered from the nares [29]–[31]. For example, in one study of 371 nasal *S. aureus* isolates, only 7 (1.9%) were resistant to trimethoprim-sulfamethoxazole [29]. Our data are not in agreement with another study reporting trimethoprim-sulfamethoxazole susceptibility rates of 78.6% and 95.3%, for MRSA and MSSA isolates, respectively, recovered from patients in 12 cities across China [30]. Similar to other surveillance studies [13], [30], we found high rates of resistance for nasal *S. aureus* isolates to penicillin, ampicillin/sulbactam and erythromycin, and no discernable resistance of these isolates to vancomycin, quinupristin/dalfopristin or linezolid. Mupirocin is used commonly for treating MRSA skin and soft-tissue infections and eliminating nasal MRSA colonization among patients and medical staff. Our study indicated that 10.7% of nasal MRSA isolates were resistant to mupirocin, in agreement with mupirocin resistance rates observed for MRSA isolates recovered in the context of clinical infection in another study conducted in Wenzhou, China [31]. Therefore, different decolonization strategies including mupirocin may prove more successful for reducing nasal MRSA carriage among members of the Chinese medical community, and possibly Chinese hospital-associated MRSA infections.

Understanding the molecular characteristics of MRSA isolates recovered from healthy individuals may facilitate a better understanding of mechanisms for geographic distribution of MRSA in certain populations, as well as the population-based risk for aggressive community-acquired MRSA infections. CA- MRSA isolates from different geographic areas have demonstrated significant genetic diversity [12]. The vast majority of CA-MRSA isolates worldwide belong to 5 clones: pandemic clone USA300 (ST8), European clone (ST80), midwest clone (ST1), southwest pacific oceanic clone (ST30) and pacific clone (ST59) [12]. Studies performed in Taiwan indicate that ST59 was the most common MLST type for MRSA isolates associated with community-acquired infections and nasal colonization, particularly pediatric infections [29], [32], [33], [34]. The majority (90%) of MRSA isolates recovered from healthy individuals over a 5-year period in Taiwan were clustered into minor variants of two clonal types, ST59 and ST338 [29]. However, the nasal MRSA isolates recovered in our study showed considerable heterogeneity, with ST59 and ST338 accounting for only 14.3% and 3.6% of STs, respectively. One recently published study found that among MRSA isolates associated with pediatric infections on the Chinese mainland, ST59-MRSA-SCCmecVI-t437 was the most common clone identified (59%), followed by ST1 (8%) and ST338 (8%) [35]. ST59-MRSA-SCCmecVI-t437 and ST338-MRSA-SCCmecV-t163 clones were identified from healthy volunteers in our study, indicating that these two clones may contribute to community infection and dissemination among hospital employees. HA-MRSA infections are caused by a relatively small number of epidemic MRSA clones [36], and ST239-MRSA-SCC*mec* type III and ST5-MRSA-SCC*mec* type II are two major epidemic MRSA clones identified in China [14]. Interestingly, these two clones were not isolated in our study. In previous studies published by our group [37], [38], ST88 was the most common clone identified among PVL-positive isolates. In the present study, ST88-MRSA-SCCmecV was identified for only one PVL-negative nasal MRSA isolate. Whether CA-MRSA clones identified within the nares of healthy individuals are responsible for clinical infections caused by CA-MRSA in China remains to be determined.

CA-MRSA pathogenesis has been linked to carriage of PVL encoding genes [39]. Relatively high prevalences of PVL gene expression were also found among nasal *S. aureus* isolates in Chengdu, China (22%) [41], and Taiwan (19.1%) [40], [41]. We noted a somewhat lower prevalence (11%) of PVL gene expression for nasal MRSA isolates in our study. Whether a higher prevalence of PVL expression for nasal *S. aureus* isolates portends a higher rate of clinical *S. aureus* infections, and more severe CA-MRSA infections, in Chinese populations remains to be determined. Epidemiologic studies tracking incident CA-MRSA infections in our institution, and determining whether infections caused by PVL-expressing CA-MRSA isolates arise in persons in contact with healthy volunteers in our study harboring these isolates, is ongoing.

ST398-MRSA is usually associated with animal infection, especially in pigs [42], and persons in close contact with animals are more likely to harbor ST398-MRSA isolates [37], [43], [44], [45]. Recently published data suggest that human infections caused by MRSA-ST398 are increasing [37], [43], [44], [45]. One nasal MRSA isolate belonging to ST398-MRSA-V-*spa* t571 was found in our study. It would be interesting to determine whether medical students performing cadaveric procedures using animals are more likely to harbor ST398-MRSA.

In conclusion, we found a relatively high prevalence of nasal MRSA carriage within healthy persons from a Chinese medical college campus. Among these nasal MRSA isolates, epidemic clones associated with community infection were found, supporting policies for reducing nasal carriage in order to prevent spread of MRSA to community members and hospitalized patients. Moreover, antibiotic resistance patterns for these isolates indicate that unique decolonization strategies may be needed.
